# Ectopic ACTH Syndrome Caused by Pheochromocytoma: A Case Report and Systematic Review

**DOI:** 10.2174/0118715303372676250628161600

**Published:** 2025-07-09

**Authors:** Jiadian Huang, Cuiping Lin, Lixuan Fang, Lijuan Gu, Xia Cai, Haixia Zeng, Kang Chen, Yiming Mu, Jianping Liu

**Affiliations:** 1 Department of Endocrinology and Metabolism, The Second Affiliated Hospital, Jiangxi Medical College, Nanchang University, Nanchang 330006, China;; 2 Department of Endocrinology, People’s Hospital of Fengcheng of Jiangxi Province, 331100, China;; 3 Department of Endocrinology, Chinese PLA General Hospital, Beijing Province 100039, China;; 4 Institute for the Study of Endocrinology and Metabolism in Jiangxi Province, Nanchang City, Jiangxi Province, 330006, China

**Keywords:** Ectopic adrenocorticotropic hormone (ACTH) syndrome, pheochromocytoma, cortisol, adrenal, metabolic alkalosis, pathological examination

## Abstract

**Introduction:**

Ectopic adrenocorticotropic hormone (ACTH) syndrome caused by pheochromocytoma is extremely rare, and its highly variable clinical manifestations make diagnosis challenging.

**Case Presentation:**

This report describes a case of a 48-year-old female with typical Cushingoid features who complained of edema in both lower limbs for 20 days. Laboratory testing showed hypokalemia with metabolic alkalosis, high levels of cortisol and ACTH, and the absence of circadian rhythm. Neither low-dose or high-dose dexamethasone suppression testing could inhibit cortisol secretion. Abdominal computed tomography showed a 32-mm mass in the left adrenal gland and hyperplasia of the right adrenal gland. The patient underwent resection of the left-sided adrenal adenoma, and pathological examination revealed features of a pheochromocytoma. Immunohistochemistry showed positivity for chromogranin A, synaptophysin, neuron-specific enolase, and ACTH, with a Ki67 labeling index of approximately 1%. Post-operatively, the patient’s electrolyte, serum cortisol, and ACTH levels returned to normal rapidly, and the symptoms of edema resolved. The diagnosis was ectopic ACTH syndrome caused by pheochromocytoma.

**Conclusion:**

Based on this case and the presented literature review, we recommend that all patients with an elevated ACTH level and adrenal mass be screened for ectopic ACTH syndrome caused by pheochromocytoma.

## 1 INTRODUCTION

Cushing’s syndrome (CS) is an endocrine disorder involving exposure of the body to excessive levels of cortisol. The estimated incidence of CS is 0.7~2.4 cases per million people per year [1], and the typical clinical manifestations of CS include abdominal obesity, impaired glucose metabolism, protein metabolism disorders, musculoskeletal disorders, and other related conditions. Endogenous CS is classified into two main types according to the patient’s adrenocorticotropic hormone (ACTH) level: ACTH-dependent (70%-80% of cases) and ACTH-independent (20%- 30% of cases). The ACTH-dependent type includes Cushing’s disease (CD) (60%-70% of ACTH-dependent cases) and ectopic ACTH syndrome (EAS) (10%-20% of ACTH-dependent cases) [2]. Patients with EAS often experience severe metabolic symptoms, hypokalemic alkalosis, and peripheral edema due to hypercortisolemia and abnormal mineralocorticoid levels. The most common neoplastic origins of EAS are small cell lung cancer, bronchial carcinoid, thymic carcinoid, islet cell carcinoma, medullary thyroid carcinoma, and pheochromocytoma, as the origin of EAS is rare (3%-5% of EAS cases). Pheochromocytoma is a tumor that arises from the adrenal medulla, and the estimated annual incidence of pheochromocytoma is 2 to 8 per million [3]. Pheochromocytoma is believed to mainly result in elevated catecholamine levels or abnormal adrenal function, leading to hyperadrenergic symptoms such as hypertension, headache, palpitation, and sweating. The combined effects of increased cortisol and increased catecholamine secretion are associated with rapid disease progression and serious life-threatening effects, conferring pheochromocytoma patients with a higher risk of death. Thus, early diagnosis is critical. Unfortunately, a recent study found that only 17% of patients with pheochromocytoma present with typical symptoms [4]. Therefore, comprehensive strategies for diagnosing this condition are needed to facilitate timely intervention and improve patient outcomes.

Currently, the clinical data on EAS due to pheochromocytoma are limited, with fewer than 100 cases reported in the literature. The present report describes a case treated recently in our hospital and summarizes the clinical characteristics of EAS caused by pheochromocytoma based on a literature review, aiming to improve clinicians’ understanding of this condition in support of its proper diagnosis.

## 2 CASE REPORT

A 48-year-old female presented with a 20-day history of bilateral lower limb edema. Her serum potassium levels ranged from 2.27-2.74 mmol/L at a local hospital and did not normalize with potassium supplementation. Detection of elevated cortisol and ACTH levels, along with normal catecholamine levels, led to the consideration of CS, resulting in the patient’s admission to our hospital. Physical examination showed stable vital signs and a body mass index (BMI) of 19.4 kg/m^2^. The patient had features of a “full moon” face, hirsutism, thin skin, scattered ecchymosis, slender upper limbs, moderate pitting edema in both lower limbs, and no striae. She had no history of hypertension, renal disease, or glucocorticoid use. She was in the perimenopausal period and had been taking estradiol/dydrogesterone for more than 3 months. Laboratory tests showed a serum potassium level of 2.46 mmol/l, and arterial blood gas analysis suggested metabolic alkalosis (Table **1**). We detected elevated levels of alanine aminotransferase. Additionally, sugar markers associated with cancer metabolism, such as CA125, CA50, and CA242, were observed. Moreover, decreased globulin and eosinophil levels were observed. Thyroid function tests indicated hypothyroidism with negative autoantibodies, suggesting central hypothyroidism. Glucose metabolism was normal, and plasma renin activity was suppressed (Table **2**). Hypercortisolemia with an abnormal circadian rhythm was noted, with a plasma ACTH concentration of 359.40 pg/ml and a 24-hour urinary cortisol level elevated to three times the normal limit (Table **2**). A low-dose dexamethasone suppression test (LDDST) failed to suppress cortisol secretion, and the elevated ACTH levels indicated ACTH-dependent CS. Magnetic resonance imaging showed a normal pituitary gland, ruling out a microadenoma (Figs. **S1A**, **S1B**). A subsequent high-dose dexamethasone suppression test (HDDST) also failed to suppress cortisol secretion, and the 24-h urine free-cortisol (UFC) level was 6237.92 µg/24 h (Table **2**), suggesting EAS. Adrenal computed tomography (CT) showed a suspected adenoma in the left adrenal region, with a plain CT value of 20 Hounsfield units (HU) in the center of the mass and an enhanced CT value of 42 HU (Figs. **1A** and **1B**), along with hyperplasia of the right adrenal gland (Fig. **1C**). Whole-body positron emission tomography (PET)/CT at the previous hospital showed a low-density shadow on the left adrenal gland, which was considered to be a functional adenoma, as well as right adrenal hyperplasia. Due to the rarity of EAS cases originating from adrenal adenomas in clinical practice and the uncertain nature of the adenoma, the preliminary diagnosis was ACTH-dependent CS with a left adrenal adenoma. The patient was given sufficient potassium chloride and spironolactone treatment (potassium chloride 1 g BID and spironolactone 40 mg TID), but the low serum potassium level persisted. The patient exhibited fatigue, poor appetite, edema in both lower limbs, and a poor overall condition. After multidisciplinary consultation and full communication with the patient, she declined bilateral inferior petrosal sinus sampling for further diagnosis and chose to undergo left adrenal adenoma resection. Laparoscopic left adrenal tumor resection was performed 3 weeks later and was successful. Postoperatively, the patient experienced transient hypotension that resolved with blood volume replacement and continued potassium supplementation. On the second postoperative day, her serum potassium level was 2.84 mmol/L, and reductions in serum cortisol to 39.14 µg/dl and plasma ACTH to 64.11 pg/ml were observed (Table **3**). Her electrolyte, cortisol, and ACTH levels returned to normal values by postoperative day 5 (Table **3**), and the edema symptoms in both lower limbs were significantly relieved.

The excised left adrenal adenoma was found to originate from the adrenal cortex. Pathological evaluation showed a single well-circumscribed, solid, homogenous mass (27 × 22 × 20 mm^3^) with a gray-red cut surface (Fig. **S2**). Hematoxylin and eosin staining revealed the following: tumor cells arranged in a strip-and-nest shape, abundant blood sinuses, and abundant cytoplasm in the tumor cells (Fig. **2A**). Immunohistochemical staining was positive for chromogranin A (CgA), synaptophysin (Syn), neuron-specific enolase (NSE), and ACTH and negative for creatine kinase (CK), vimentin (Vim), and S100. The percentage of cells with p53 staining was approximately 3%, and the Ki67 labeling index was approximately 1% (Figs. **2B** and **2C**). The pathological diagnosis of the left-sided adrenal tumor was pheochromocytoma. The patient was diagnosed with EAS from pheochromocytoma. Remarkably, her blood pressure remained normal throughout the disease and treatment, and she experienced no pheochromocytoma-specific symptoms, such as headache, palpitation, or sweating. One month after discharge, the patient’s Cushingoid symptoms had improved, and her electrolyte, cortisol, and ACTH levels were normal when measured in the local hospital.

## 3 DISCUSSION

This report presents a rare clinical case of CS secondary to ectopic ACTH production by pheochromocytoma. In addition to the rarity of this etiology, the specific characteristics of this case in the context of previously reported cases are worth discussing.

A unique feature of the present case is that the patient’s blood pressure remained normal, and she had no pheochromocytoma-specific symptoms. Upon reviewing the literature, we found that most patients usually have elevated blood pressure, as both pheochromocytoma and EAS can cause hypertension. We speculate that the main reason why the present case of pheochromocytoma did not cause hypertension was the production of fewer catecholamines or high levels of vasodilator hormones, such as vasoactive intestinal peptides and neuropeptide Y, resulting in no obvious increase in blood pressure. In addition, it is also possible that the patient had an asymptomatic (*i.e.*, non-catecholamine-secreting) pheochromocytoma. According to the latest guidelines, pheochromocytoma in adrenal masses cannot be excluded even in the absence of biochemical evidence of catecholamine excess [5]. The diagnosis of EAS was made in this case based on the results of biochemical and imaging examinations. We initially did not consider pheochromocytoma, and thus, did not measure the catecholamine levels (plasma-free metanephrines) as recommended by the Endocrine Society Clinical Guidelines Subcommittee (CGS) [5]. The absence of biochemical evidence in favor of pheochromocytoma increased the difficulty of the diagnosis. Furthermore, the omission of postoperative catecholamine level monitoring limited our assessment of surgical recovery, and this deficiency deserves attention for precise diagnosis in similar cases.

The patient in the present case did show characteristics of recurring severe hypokalemia due to the mineralocorticoid effect of hypercortisolism. Hypokalemia results from urinary losses induced by the mineralocorticoid effect of cortisol. Under normal circumstances, the mineralocorticoid effect of cortisol is insignificant due to local conversion to cortisone *via* the action of 11 beta-hydroxysteroid dehydrogenase (11β-HSD) [6]. In patients with EAS, excessive cortisol saturates the action of 11β-HSD and leads to the appearance of mineralocorticoid action of cortisol [7, 8]. However, in other typical cases of EAS caused by pheochromocytoma, elevated catecholamines cause insufficient peripheral vascular volume by constricting blood vessels, activating the renin-angiotensin-aldosterone system (RAAS), increasing renin and aldosterone secretion, and further reducing blood potassium levels. A possible mechanism for the inhibition of renin activity in the present case involves cortisol exerting its mineralocorticoid effect on the convoluted/collecting distal tubules, creating a state of apparent mineralocorticoid excess with suppression of renin and aldosterone secretion [8, 9]. As a result, renin activity decreased, and aldosterone remained in the normal low range. Thus, from a pathophysiological standpoint, the most effective management strategy for hypokalemia is to effectively treat the hypercortisolism itself. On the other hand, the RAAS system could be inhibited in severe hypokalemia, resulting in decreased renin and aldosterone secretion.

From our literature review, most patients with EAS from pheochromocytoma exhibit abnormal glucose metabolism due to cortisol-induced hyperglycemia *via* heightened insulin resistance, which hinders a compensatory rise in pancreatic β cells. However, the present case represents an exception, because the patient’s plasma glucose level remained normal. This case also exhibited biochemical evidence of central hypothyroidism, which is considered to be reversible central hypothyroidism caused by EAS. The responsible mechanism includes cortisol reducing the secretion of serum thyroid-stimulating hormone (TSH) by reducing the expression of thyroid-releasing hormone mRNA in the hypothalamus [10]. Cortisol can also induce a decrease in deiodinase activity and reduce the level of thyroid hormone-binding globulin [11]. Generally, thyroid hormone levels gradually return to normal after adrenalectomy. Measurement of gonadal hormones in the present case showed increased follicle-stimulating hormone (FSH) and estradiol (E) levels and reduced luteinizing hormone (LH) and progesterone (P) levels. These results were associated with the patient’s perimenopausal status and estradiol/dydrogesterone supplementation, which represent normal physiological changes in a 48-year-old woman in the early menopausal transition, a period marked by a declining follicle cohort and reduced inhibin B levels, leading to a loss of pituitary inhibition and increased FSH secretion. The higher E level in this case was mainly related to exogenous estradiol/dydrogesterone supplementation. Due to a prolonged follicular phase and luteal dysfunction that occur during perimenopause, LH levels are low, resulting in reduced P secretion [12]. Elevated ACTH levels stimulate the secretion of adrenal androgens from the adrenal cortex, leading to increased T and dehydroepiandrosterone sulfate (DHEAS) levels.

Our literature review of cases of ACTH-producing pheochromocytomas identified 70 cases reported in the English language literature, including the present case (Table **4**) [13-74]. From the review of these cases in consideration of the present case, the clinical characteristics of EAS caused by pheochromocytoma can be summarized as follows: 1) short onset time with nontypical symptoms and Cushingoid symptoms and/or pheochromocytoma-related manifestations [44, 54, 75]; 2) female predominance and common involvement of left-side adrenal adenomas, with hyperplasia possible in the right adrenal gland [58, 75]; 3) significantly increased cortisol and ACTH levels [43, 55, 75]; 4) inability of LDDST and HDDST to inhibit cortisol secretion [75, 76]; 5) increased catecholamine and metabolite levels [43, 63]; 6) positivity for ACTH and CgA on pathological examination [51, 58, 76]; 7) secretion of corticotropin-releasing hormone (CRH), vasoactive intestinal peptide, calcitonin, somatostatin, and others in addition to ACTH and catecholamine [77-79]; 8) frequent and serious hypokalemic metabolic alkalosis, hypertension, and hyperglycemia [9, 58]; 9) occurrence secondary to infection, venous thrombosis, mental disorders, *etc* [6, 75]; 10) combination with reversible central hypothyroidism [54, 58, 64]; 11) rapid return of cortisol and ACTH to normal levels after adrenalectomy with significant relief of symptoms [62, 64]; and 12) benign tumors with good prognosis and short-term need for hormone replacement therapy [46, 55, 58].

Currently, adrenalectomy is the preferred treatment for EAS from pheochromocytoma. Unlike other ACTH-producing ectopic tumors, most pheochromocytomas are benign, allowing curative outcomes post-unilateral adrenalectomy. Bilateral adrenalectomy is an option for exceptionally severe symptoms or reciprocal adrenal tumors. Preoperative management involves the administration of alpha-blockers and antihypertensives to normalize blood pressure. Use of adrenocorticotropic hormone synthetase inhibitors, such as ketoconazole, mitotane, and metyrapone, or the use of melatonin supplementation [80, 81], may be considered to suppress cortisol secretion if needed, contingent upon the clinical scenario. Before surgical treatment, patients’ cortisol levels should be closely monitored, and drug doses should be adjusted early enough to control the level of cortisol. In clinical practice, prophylactic oral anticoagulation therapy should be used to prevent venous thromboembolism based on the patient’s risk. In addition, symptomatic treatment, such as potassium supplementation and hypoglycemic therapy, should be given. Postoperatively, patients usually need short-term hormone replacement therapy. According to the guidelines of the Society of Endocrinology [5], in patients with pheochromocytoma, the mutations in susceptibility genes may be large. Genetic testing can be performed using an 11-gene panel (*FH*, *MAX*, *NF1*, *RET*, *SDHAF2*, *SDHA*, *SDHB*, *SDHC*, *SDHD*, *TMEM127*, and *VHL*), especially for young patients with a relevant family history. Simultaneously, genetic testing is also beneficial for differentiating non-catecholamine-secreting pheochromocytomas with a higher malignant propensity. The patient in this case did not have a relevant family history and declined to undergo genetic testing, which represents a limitation of this case report. Genetic testing in future cases could provide beneficial information. Overall, when high FDG-PET uptake is accompanied by an SDHB mutation, the likelihood of a malignant pheochromocytoma should be carefully considered.

## CONCLUSION

We present a challenging case of EAS caused by a pheochromocytoma. Initially, the patient presented with typical CS manifestations, with a high level of ACTH but no manifestations of a high catecholamine level, such as hypertension and hyperglycemia, which increased the difficulty in diagnosing this case. Through active treatment of symptoms and comprehensive imaging and histological examinations, the source of ACTH was finally determined. Pheochromocytoma is a rare source for EAS, and the clinical manifestations of such cases are nontypical, making the diagnosis of these cases challenging.

## Figures and Tables

**Fig. (1):**
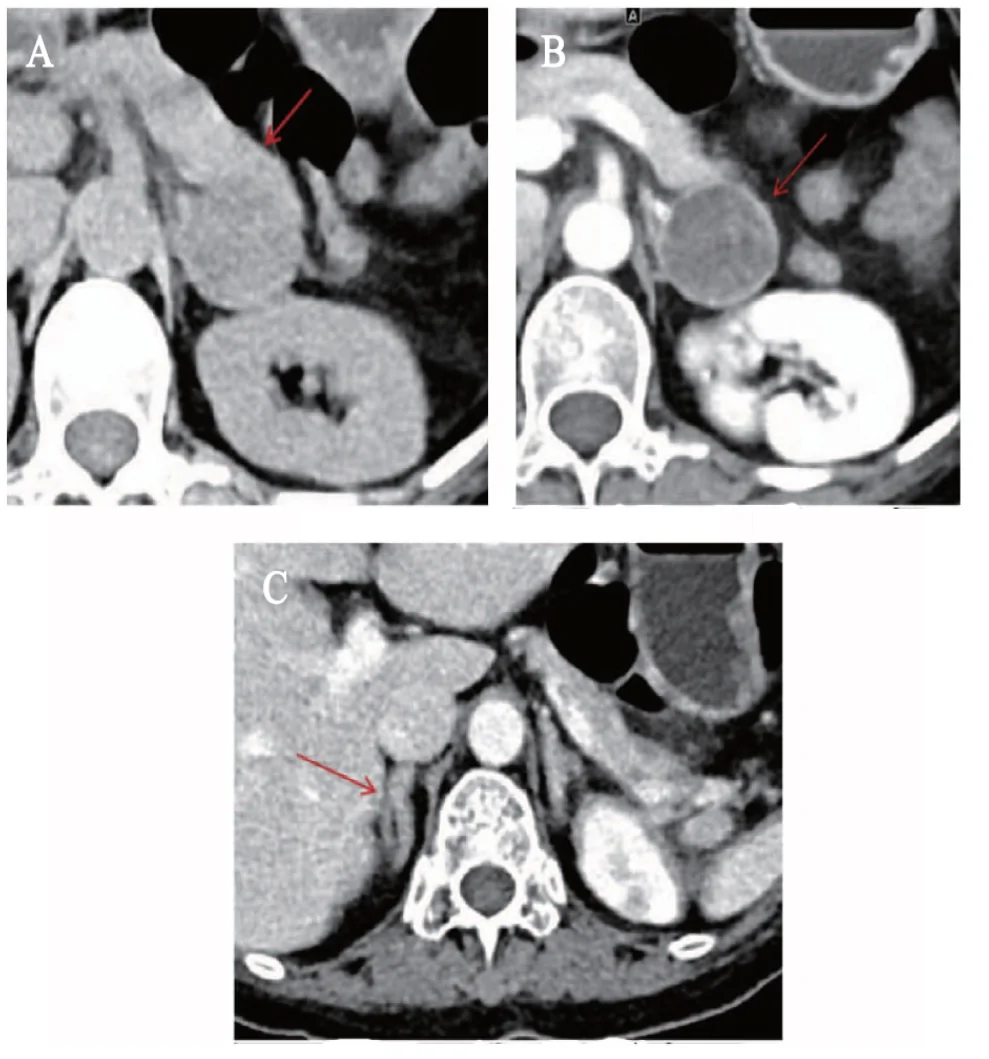
Adrenal CT scans showing a suspected adenoma in the left adrenal region. (**A**) Plain CT scan. The red arrow points to a mass ~32 mm in diameter found in the left adrenal gland. (**B**) Enhanced CT scan of cross-sectional view. The red arrow points to a left adrenal mass with mild enhancement that was suspected to be an adenoma. (**C**) Enhanced CT scan of cross-sectional view. The red arrow points to the right adrenal hyperplasia.

**Fig. (2):**
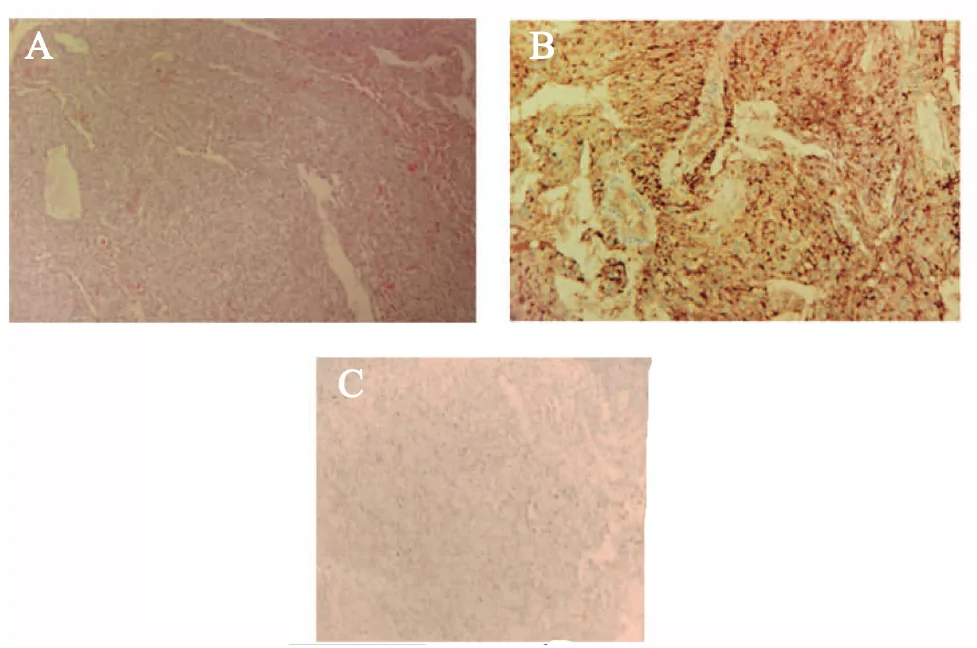
Immunohistochemical staining of excised tumor tissue. (**A**) Hematoxylin and eosin staining; (**B**) p53 staining, and (**C**) Ki67 labeling.

**Table 1:** Laboratory test results at presentation.

**Test**	**Result**	**Reference Range**
WBC count	7.82/L	(3.5-9.5)×10^9/L
RBC count	4.24/L	(3.8-5.1)×10^12/L
Hb	137 g/L	110-150 g/L
PLT count	136/L	(125-350)×10^9/L
Neutrophil count	7.24/L	(2-7)×10^9/L
Eosinophil count	0.00/L	(0.1-0.4)×10^9/L
TP	58.89 g/L	55-85 g/L
GLB	17.76 g/L	20-40 g/L
ALB	41.13 g/L	40-55 g/L
K^+^	2.46 mmol/L*	3.5-5.3 mmol/L
Na^+^	144.18 mmol/L	137-147 mmol/L
AST	24.09 U/L	13-35 U/L
ALT	57.84 U/L*	7-40 U/L
Cr	45.79 μmol/L	41-73 μmol/L
eGFR	134.40 mL/min/1.73m^2^	≥90 mL/min/1.73m^2^
Plasma glucose	8.37 mmol/L	<11.1 mmol/L
HbA1c	5.4%	<6.5%
TG	0.77 mmol/L	<1.7 mmol/L
TC	4.94 mmol/L	<5.18 mmol/L
pH	7.59	7.35-7.45
PaCO_2_	30.5 mmHg*	35.0-45.0 mmHg
BE	7.4 mmol/L	-2-3 mmol/L
HCO_3_^-^	29.1 mmol/L*	22-26 mmol/L
CRP	0.6 mg/L	0-10 mg/L
CA125	100.90 U/mL*	0-35 U/mL
CA50	26.00 IU/mL*	<25 IU/mL
CA242	23.35 IU/mL*	<10 IU/mL
NSE	3.71 mg/mL	<10 ng/mL

**Abbreviations:** WBC, white blood cell; RBC, red blood cell; Hb, hemoglobin; PLT, platelet; TP, total protein; GLB, globulin; ALB, albumin; K^+^, potassium; Na^+^, sodium; ALT, alanine aminotransferase; AST, aspartate aminotransferase; Cr, creatinine; eGFR, estimated glomerular filtration rate; HbA1c, hemoglobin A1c; TG, triglycerides; TC, total cholesterol; BE, base excess; HCO_3_^-^, bicarbonate; CRP, C-reactive protein; CA125, cancer antigen-125; CA 50, cancer antigen-50; CA242, cancer antigen-242; NSE neuron-specific enolase. **Notes:** Routine blood testing was performed using the five classification method (SYSMEX XN-9000); carbohydrate antigens were measured by chemiluminescence immunoassay (Roche cobas eb02); HbA1c was measured by high performance liquid chromatography (4500MD, AB SCIEX, USA); and blood lipids, liver and kidney function, and electrolytes were measured by automatic biochemical analyzer (Beckman Coulter AU5831, USA). Blood gas analysis was performed upon admission. All remaining biochemical indices were measured in a venous blood sample drawn in the early morning after fasting for more than 8 hours. *Indicates values outside the reference range.

**Table 2:** Endocrine profile.

**Test**	**Result**	**Reference Range**
FT_3_	2.16 pg/mL*	2.3-4.2 pg/mL
FT_4_	1.27 ng/mL	0.89-1.8 ng/dL
TSH	0.028 mIU/L*	0.55-4.78 mIU/L
FSH	49.15 mIU/mL*	5.8-21 mIU/mL
LH	4.37 mIU/mL*	15.9-54 mIU/mL
E	55.43 pg/mL*	0-32.2 pg/mL
P	1.23 ng/mL*	3.3-26 ng/mL
PRL	12.24 ng/mL	2.8-29.2 ng/mL
T	78.40 ng/dL*	14-76 ng/dL
DHEA-S	590 µg/dL*	19-231 µg/dL
PAC (supine position)	3.03 ng/dL	3.0-16.0 ng/dL
PRA (supine position)	0.01 ng/mL/h*	0.15-2.33 ng/mL/h
ARR (supine position)	303*	<30
**Serum cortisol** 8 AM 4 PM 0 AM	130.14 µg/dL* 118.18 µg/dL* 100.56 µg/dL	4.3-22.4 µg/dL 3.09-16.66 µg/dL
**Plasma ACTH** 8 AM 4 PM 0 AM	359.40 pg/mL* 395.20 pg/mL* 247.20 pg/mL*	6.0-40 pg/mL 3.0-30 pg/mL <20 pg/mL
24-h UFC	9973.92 µg/24 h	19.3~317.53 µg/24 h
**LDDST** Serum cortisol (8 AM)	116.22 µg/dL*	<1.8 µg/dL
**HDDST** Serum cortisol (8 AM) 24-h UFC	118.19 µg/dL 6237.92 µg/24 h	<50% of the baseline value <50% of the baseline value

**Abbreviations:** FT_3,_ free triiodothyronine; FT_4,_ free thyroxine; TSH, thyroid-stimulating hormone; FSH, follicle-stimulating hormone; LH, luteinizing hormone; E, estradiol; P, progesterone; PRL, prolactin; T, testosterone; DHEA-S, dehydroepiandrosterone-sulfate; PAC, plasma aldosterone concentration; PRA, plasma renin activity; ARR, aldosterone-to-renin ratio; UFC, urine free cortisol; LDDST, low dose dexamethasone suppression test; HDDST, high dose dexamethasone suppression test. **Notes:** The above indicators were measured by chemiluminescence immunoassay (Roche cobas eb02). 24-h UFC: On the morning of Day 1, urine was voided and discarded. Then all urine excreted in 24 hours was collected into one container until the same time on the morning of Day 2. The total 24-h urine volume was recorded, and the sample was mixed well before 5-10 ml of urine was taken for cortisol level testing. For analysis of the cortisol rhythm, the serum cortisol level was measured at 8:00, 16:00, and 0:00 during the examination, but the venous blood draw at midnight had to be completed within 1-3 minutes after waking the patient with avoidance of multiple puncture stimulation. ACTH was measured simultaneously. For the LDDST: Dexamethasone was administered orally at a dose of 0.5 mg, once every 6 h for 2 consecutive days. Then the serum cortisol levels were measured before and after the administration of medication for comparison. Alternatively, 24-h urine samples were collected before administration of the medication and on the second day of medication administration separately, for determination of the urinary free cortisol (UFC) level. For the HDDST: The method was the same as that for the LDDST, but the oral dose of dexamethasone was adjusted to 2 mg, administered once every 6 h for 2 days (*i.e*., 8 mg/day × 2 days). *Indicates values outside the reference range.

**Table 3:** Postoperative laboratory results.

**Postoperative Timing**	**Test**	**Result**	**Reference Range**
**Postoperative day 2**	K^+^	2.84 mmol/L	3.5-5.3 mmol/L
	**Serum cortisol** 8 AM 0 AM	39.14 µg/dL 7.22 µg/dL	4.3-22.4 µg/dL
	**ACTH** 8 AM	64.11 pg/mL	6.0-40 pg/mL
**Postoperative day 5**	K^+^	4.32 mmol/L	3.5-5.3 mmol/L
	**Serum cortisol** 8 AM 0 AM	16.82 µg/dL 13.24 µg/dL	4.3-22.4 µg/dL
	**Plasma ACTH** 8 AM	18.11 pg/mL	6.0-40 pg/mL

**Notes:** The detection methods were the same as described in Table **2**.

**Table 4:** Characteristics of reported cases of EAS due to pheochromocytoma.

**Author/Year**	**Age, y**	**Sex**	**Location, Size**	**Cushingoid Appearance**	**Hypertension**	**Hyperglycemia**	**Hypokalemia**	**Sympathetic Symptoms**	**Symptom Duration**	**Positive Immunostaining**
Meloni CR *et al*. (1966) [13]	51	F	Right, N/A	+	+	+	+	+	6 m	ACTH
Spark RF *et al*. (1979) [14]	47	F	Left, 30 mm	+	+	+	+	+	1 y	ACTH
Forman BH *et al*. (1979) [15]	51	F	Right, 80×50 mm	+	+	+	+	+	3 m	ACTH
Schroeder JO *et al*. (1984) [16]	47	F	Left, 30 mm	+	+	+	+	+	8 w	NSE
Kakudo K *et al*. (1984) [17]	54	F	Left, 110×110×60 mm	-	+	-	-	+	N/A	N/A
Lamovec J *et al*. (1984) [18]	42	F	Left, 28×23×21 mm	+	+	N/A	N/A	+	3 y	ACTH, NSE
Bruining HA *et al*. (1985) [19]	38	F	Left, 60 mm	+	+	+	+	+	N/A	N/A
Beaser RS *et al*. (1986) [20]	36	F	Right, 40×32×15 mm	+	+	+	+	+	9 m	ACTH
Jessop DS *et al*. (1987) [21]	61	M	Left, N/A	+	+	N/A	N/A	N/A	N/A	N/A
Sakurai H *et al*. (1987) [22]	44	F	Right, 75×75×95 mm	+	+	+	+	+	4 m	ACTH
O'Brien T *et al*. (1992) [23]	49	F	Left, 34×33×30 mm	+	+	+	-	-	1 m	CgA, Syn, NSE, ACTH, CRH
Terzolo M *et al*. (1994) [24]	35	F	Left, 35 mm	+	+	+	+	-	4 m	CgA, ACTH, NSE
Chen H *et al*. (1995) [25]	51	F	Left, 30 mm	+	+	+	+	+	N/A	ACTH
Chen H *et al*. (1995) [25]	38	F	Right, 25 mm	+	+		-	+	N/A	ACTH
Chen H *et al*. (1995) [25]	26	M	Left, 30 mm	+	+	+	+	-	N/A	N/A
Chen H *et al*. (1995) [25]	57	F	Right, 28 mm	-	+	+	-	-	N/A	ACTH
Loh KC *et al*. (1996) [26]	25	F	Left, 40 mm	+	+	N/A	N/A	+	6 w	ACTH
Sato M *et al*. (1998) [27]	41	F	Left, 60 mm	-	-	+	-	+	3 m	ACTH, NSE
Finkenstedt G *et al*. (1999) [28]	29	F	Left, 64×58 mm	+	+	+	N/A	-	2 y	CgA, Syn, NSE
Khalil WK *et al*. (1999) [29]	44	F	Left, 25 mm	-	+	-	N/A	-	N/A	N/A
Eng PH *et al*.(1999) [30]	24	M	Left, 77 mm	+	+	+	-	+	1 y	CgA, NSE, CRH
White A *et al*. (2000) [31]	44	F	Left, 40 mm	+	+	N/A	+	+	1 m	N/A
van Dam PS *et al*. (2002) [32]	30	M	Right, 35×45 mm	+	+	+	+	+	1 y	ACTH
Oh HC *et al*. (2003) [33]	28	F	Right, 30×50 mm	+	+	+	+	+	8 m	CgA, Syn, ACTH
Otsuka F *et al*. (2005) [34]	55	F	Left, 50 mm	+	+	+	-	+	6 m	CgA, Syn, ACTH
Danilovic DL *et al*. (2007) [35]	71	M	Left, 45 mm	-	+	+	+	N/A	4 m	CgA, Syn
Brenner N, *et al* (2008) [36]	53	F	Left, 35 mm	+	+	N/A	N/A	N/A	N/A	N/A
Nijhoff MF *et al*. (2009) [37]	74	M	Left, 35 mm	+	+	+	+	-	4 m	CgA, Syn, ACTH
Ruggeri RM *et al*. (2009) [38]	50	F	Right, 78×48×50 mm	+	+	N/A	+	-	N/A	CgA, CRH, S100
Fiebrich HB *et al*. (2009) [39]	67	M	Left, 20 mm	N/A	N/A	N/A	N/A	N/A	N/A	N/A
Ramasamy MM *et al*. (2010) [40]	30	F	Left, 35×30 mm	+	+	+	N/A	-	9 m	CgA, ACTH
Bernardi S *et al*. (2011) [41]	63	F	Right, 30×20×35 mm	-	+	+	+	+	1 m	CgA, Syn, S100, CD56, ACTH
Li XG *et al*. (2012) [42]	15	F	Right, 65 mm	+	+	-	-	-	N/A	CgA, Syn, CD56, ACTH
Ballav C *et al*. (2012) [43]	49	F	Left, 41×38 mm	+	+	N/A	+	+	2 m	ACTH
Folkestad L *et al*. (2014) [44]	70	F	Right, 30×18 mm; Left, 17×12 mm	+	N/A	+	+	N/A	N/A	CgA, Syn, NSE, S100, CD56
Folkestad L *et al*. (2014) [44]	67	F	Right, 30mm	+	+	+	-	N/A	N/A	CgA, Syn, ACTH
Zhang Y *et al*. (2015) [45]	37	F	Right, 12 mm Left, 40×30×20 mm	+	+	-	N/A	+	3 y	Right: CgA, CD56, S100 Left: CgA, Syn, S100
Flynn *et al*. (2016) [46]	63	F	Right, 10 mm	+	+	+	+	+	N/A	CgA, ACTH
Sakuma I *et al*. (2016) [47]	56	F	Left, 53×40×40 mm	+	+	+	N/A	+	N/A	CgA, Syn, ACTH
Fukasawa M *et al*. (2016) [48]	58	M	Right, 17×23 mm; Left, 28×33 mm	-	-	+	+	-	N/A	ACTH
Lwin TM *et al*. (2016) [49]	48	M	Left, 39 mm	+	+	N/A	N/A	-	3 m	CgA, Syn, S100
Lois KB *et al*. (2016) [50]	69	F	Right, 30 mm	+	+	+	+	+	2 w	CRH
Lois KB *et al*. (2016) [50]	64	F	Left, N/A	-	+	+	-	+	N/A	CgA, ACTH
Lois KB *et al*. (2016) [50]	65	M	Right, 40 mm	-	+	+	-	-	N/A	CRH
Chen F *et al*. (2016) [51]	58	M	Left, 48 mm	+	+	+	+	-	2 m	CgA, Syn, ACTH, S100
Winter EM *et al*. (2016) [52]	58	F	Right, 70×70×70	-	+	N/A	-	-	N/A	ACTH
Falhammar H *et al*. (2017) [53]	50	F	Left, 35× 22×15 mm	-	+	+	+	+	3 y	ACTH
Geva GA *et al*. (2018) [54]	70	M	Right, 36×34×22 mm	-	+	+	N/A	+	1 y	ACTH, Syn
Inoue M *et al*. (2018) [55]	46	M	Right, 60mm	-	+	+	+	+	N/A	CgA, Syn, ACTH
Wang BP *et al*. (2018) [56]	27	F	Right, 60mm	+	-	+	+	-	1 m	CgA, CD56, ACTH, CRH
Clinico-pathological Conference (2018) [57]	45	F	Left, 30×25×20 mm	-	+	+	+	+	2 y	CgA, Syn, ACTH
Gabi JN *et al*. (2018) [58]	46	F	Left, 38×36×38 mm	+	+	+	+	+	10 w	CgA, Syn, S100, ACTH
Wan Muhamad Hatta SF *et al*. (2019) [59]	51	F	Left, 40×30 mm	-	+	+	+	+	10 d	ACTH
Krylov V *et al*. (2020) [60]	49	F	Left, 27×30×46 mm	+	+	+	+	+	N/A	CgA, Syn, S100, ACTH
Lee MN *et al*. (2020) [61]	64	F	Right, 17 mm	-	+	+	-	+	5 y	CgA, Syn, ACTH
Zaman S *et al*. (2020) [62]	55	M	Right, 38 mm	+	+	+	+	+	4 w	ACTH
Leader N *et al*. (2021) [63]	62	M	Left, 28×29×33 mm	-	+	+	+	N/A	N/A	N/A
Kishlyansky D *et al*. (2022) [64]	43	F	Left, 32×20×24 mm	+	+	-	+	+	8 y	Syn, ACTH
Naeem Durrani M *et al*. (2022) [65]	30	F	Left, 350×250×200 mm	+	+	+	+	-	20d	N/A
Saishouji F *et al*. (2022) [66]	66	M	Left, 120mm	+	+	+	+	+	7y	TH, CBH, Cga
Z T Useinova *et al*. (2023) [67]	31	F	Left, 37x30x46 mm	-	+	+	+	+	2 y	CgA, Syn, ACTH
Ebuh V *et al*. (2023) [68]	66	M	Right, 574 × 534 mm	-	+	+	-	+	N/A	N/A
Guia Lopes ML *et al*. (2023) [69]	51	M	Left, 44 × 40 mm	+	+	+	+	-	6m	ACTH
Foppiani L *et al*. (2023) [70]	72	F	Left, 250 mm	-	+	+	+	+	6m	CgA
Omotosho YB *et al*. (2024) [71]	71	F	Right, 190 × 160 mm	+	+	+	+	+	6y	CgA, Syn, ACTH
Moctezuma S *et al*. (2024) [72]	57	F	Right, 480 × 450 × 500 mm	+	+	+	+	+	2m	N/A
Rifai K *et al*. (2024) [69]	55	F	Left, 35 mm	+	+	+	+	N/A	3y	CgA, Syn, S100
Mizutani G *et al*. (2024) [73]	63	M	Right, 600 mm	-	+	+	-	N/A	5y	CgA, Syn, ACTH
Li S *et al*. (2024) [74]	46	M	Right, 530 × 470 mm	-	+	+	-	+	N/A	N/A
Present case	48	F	Left, 27×22×20 mm	+	-	-	+	-	20 d	CgA, Syn, NSE, ACTH

**Abbreviations:** F, female; M, male; N/A, not available; y, year; m, month; w, week; ACTH, adrenocorticotropic hormone; CRH, corticotropin-releasing hormone; CgA, chromogranin A; Syn, synaptophysin; NSE, neuron-specific enolase.

## LIST OF ABBREVIATIONS

ACTH: Adrenocorticotropic Hormone
CS: Cushing’s Syndrome
CD: Cushing’s Disease
EAS: Ectopic ACTH Syndrome
LDDST: Low-Dose Dexamethasone Suppression Test
UFC: Urine-Free Cortisol
CT: Computed Tomography
PET: Positron Emission Tomography
CgA: Chromogranin A
Syn: Synaptophysin
NSE: Neuron-Specific Enolase
CK: Creatine Kinase
Vim: Vimentin
11β-HSD: 11 Beta-Hydroxysteroid Dehydrogenase
RAAS: Renin-Angiotensin-Aldosterone System
TSH: Thyroid-Stimulating Hormone
FSH: Follicle-Stimulating Hormone
E: Estradiol
LH: Luteinizing Hormone
P: Progesterone
DHEAS: Dehydroepiandrosterone Sulfate
CRH: Corticotropin-Releasing Hormone

## STANDARDS OF REPORTING

CARE guidelines were followed.
