# Supplementary material for: Ectopic ACTH Syndrome Caused by Pheochromocytoma: A Case Report and Systematic Review
Source: Endocr Metab Immune Disord Drug Targets. 2025 Jul 9;26:E18715303372676. doi: 10.2174/0118715303372676250628161600 (PMC13631853; doi:10.2174/0118715303372676250628161600)
Supplement: Supplementary file 1 [file EMIDDT-26-E18715303372676_SD1.pdf]

Supplementary Material

Ectopic ACTH Syndrome Caused by Pheochromocytoma: A Case Report and Systematic Review

Jiadian Huang[1,#], Cuiping Lin[1,#], Lixuan Fang[1,#], Lijuan Gu[2], Xia Cai[1], Haixia Zeng[1], Kang Chen[3], Yiming Mu[3] and Jianping Liu[1,4,]*

[1]Department of Endocrinology and Metabolism, The Second Affiliated Hospital, Jiangxi Medical College, Nanchang University, Nanchang 330006, China; [2]Department of Endocrinology, People's Hospital of Fengcheng of Jiangxi Province, 331100, China; [3]Department of Endocrinology, Chinese PLA General Hospital, Beijing Province 100039, China; [4]Institute for the Study of Endocrinology and Metabolism in Jiangxi Province, Nanchang City, Jiangxi Province, 330006, China

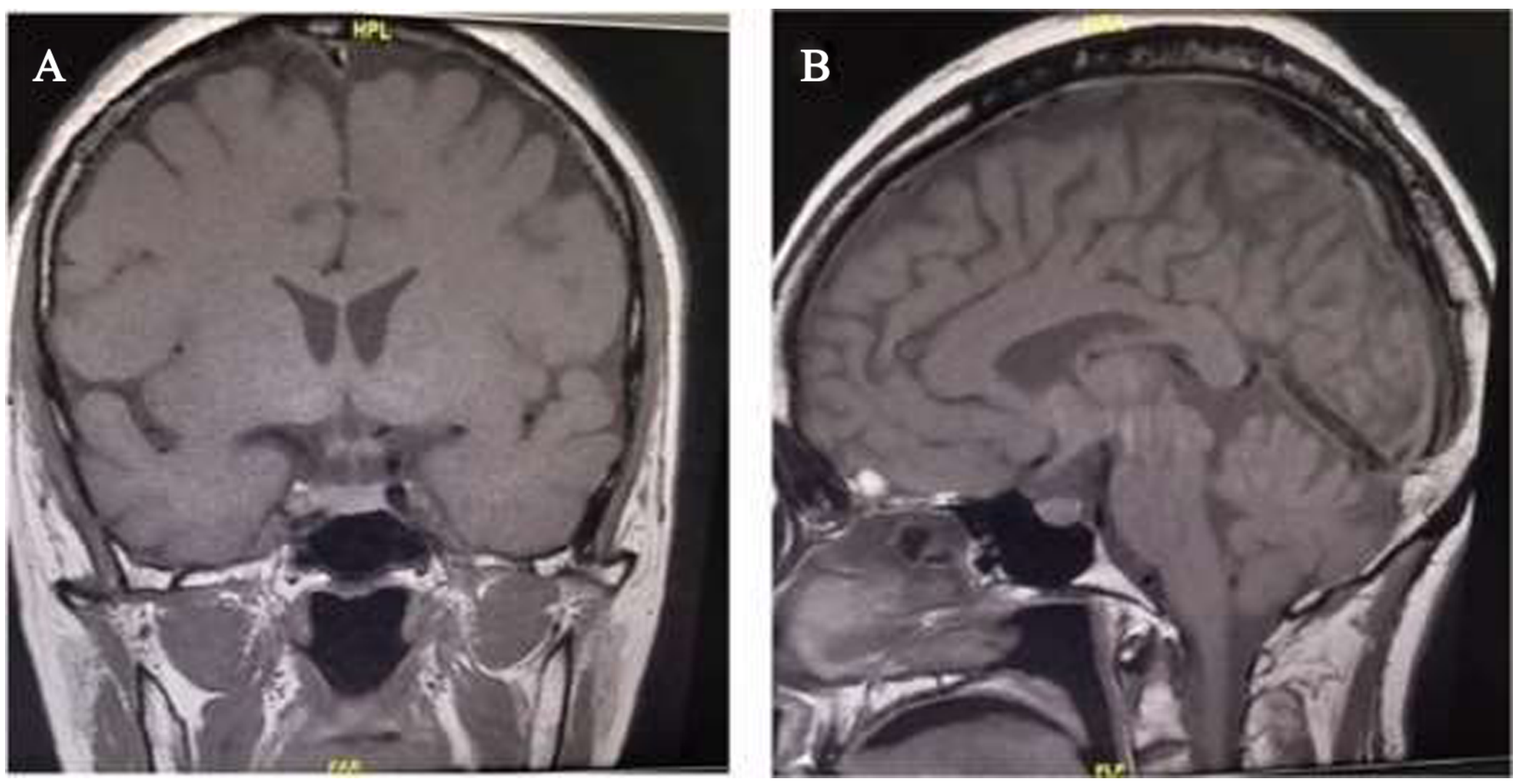

Figure S1: Magnetic resonance imaging (MRI) showing a normal pituitary gland, ruling out a microadenoma. (**A**) Coronal T1-weighted image; (**B**) Midline sagittal T2-weighted image

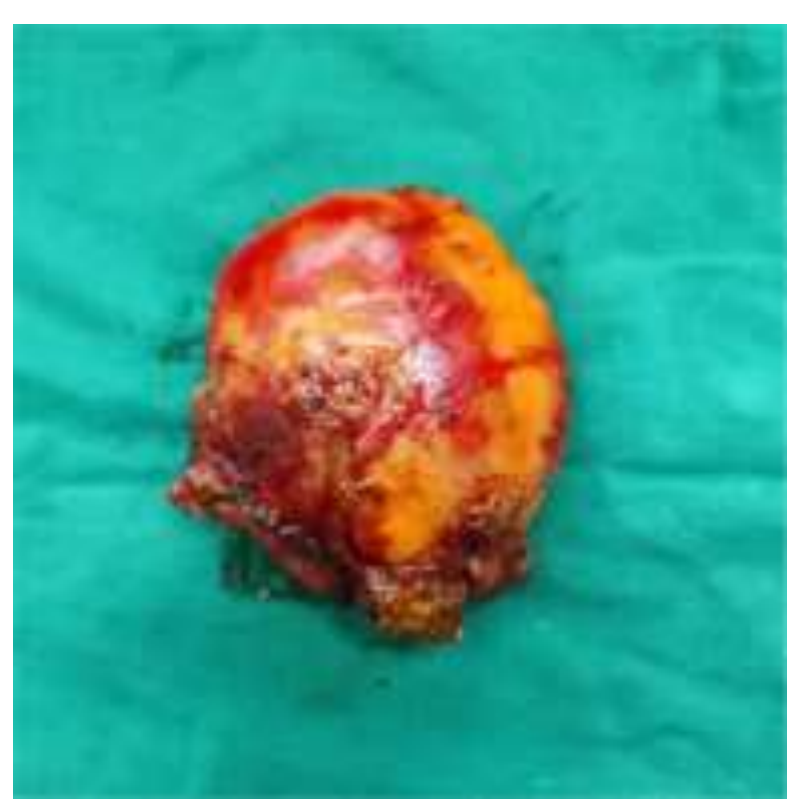

Figure S2 Photograph of excised tumor before pathological analysis. The single tumor was well-circumscribed, solid, and homogenous (27 × 22 × 20 mm^3) with a gray–red cut surface.
